# 

**Published:** 1896-06

**Authors:** 


### Society Notes.

### The North Texas Medical Association.

The North Texas Medical Association will meet in McKinney, Texas, Tuesday, Wednesday and Thursday, June 16, 17 and 18, 1896.

McKinney, Texas, May 20, 1896.

Dear Doctor:—The North Texas Medical Association will hold its next regular semi-annual meeting in the city of McKinney, Texas, beginning Tuesday, June 16, and continue its sessions for three days. The meeting will be called to order at 11 o'clock a. m. You are cordially invited to be present, and to contribute an essay, or a report of cases.

The meetings of the Association in the past have been both pleasant and profitable, and the large number of papers already received by the secretary, and others that will be ready and presented at the meeting, give promise of a most delightful scientific entertainment. Some distinguished visitors are expected to be present and contribute papers.

By united and faithful effort this Association has gradually developed and extended its scope of influence and usefulness until it now claims four hundred members. Every progressive physician in North Texas, in harmony with the code of medical ethics, should attend and join with us in an earnest and laudable effort to advance the interests of scientific medicine, to elevate and maintain its high standard, and to foster and extend the influence of this Association.

J. C. Erwin, M. D., President.

R. D. Potts, M. D., Secretary.

[Note.—A rate of 4 cents for round trip has been made by all railroads in the State. Call for a receipt from your ticket agent and present that with your ticket to the secretary for signature on arrival at McKinney.]

---

SECTION ON PRACTICE OF MEDICINE.

Acute Nephritis Following Exanthemata—By M. C. McBride, M. D., Lebanon.

Vaccination and Does it Prevent—By J. B. Stinson, M. D., Sherman.

"Pulmonary Tuberculosis," and its Treatment—By F. W. Painter, M. D., Bloomfield.

Cholera Infantum—By C. E. Fatheree, M. D., Greenville.

Some Thoughts on Therapeutics—By Jno. E. Gibson, M. D., McKinney.

Broncho Pneumonia—By E. J. Neathery, M. D., Van Alstyne.

Retinitis Albuminurica and its Bearing in the Diagnosis of Renal Diseases—By Jno. O. McReynolds, M. D., Dallas.

Diarrheal Diseases of Infancy—By B. V. Ellis, M. D., Paris.

Asthma, New Light on the Pathology and Treatment—By William R. Howard, M. D., Fort Worth.

Delayed Resolution in Pneumonia—By O. C. Buster, M. D., Pilot Point.

Diphtheritic Paralysis—By W. R. Mathers, M. D., Rock Hill.

A Case of General Peritonitis from Suppressed Menstruation —By Joe D. Becton, M. D., McKinney.

"Tuberculosis"—By W. H. Baldridge, M. D., Wylie.

Diagnosis of Valvular Diseases of the Heart—By Joe W. Largent, M. D., McKinney.

Pneumonia—By F. M. Lennard, M. D., Atlanta.

Typho-Malarial Fever—By J. G. Baldwin, M. D., Windom.

Diphtheria and its Treatment, Antitoxin—By L. Emmett Holt, M. D., New York City.

SECTION ON OBSTETRICS AND GYNECOLOGY.

Pus in the Pelvic Cavity—Cause and Treatment—By F. D. Thompson, M. D., Fort Worth.

Puerperal Eclampsia—By S. D. Moore, M. D., Van Alstyne.

Prolapus of the Uterus and its Treatment—By I. N. Devine, M. D., Panhandle.

Metritis—By W. J. Melton, M. D., Lone Oak.

Endometritis—By B. K. Corley, M. D., Greenville.

Abortion—By James R. Nichols, M. D., Greenville.

Surgical Treatment of Retro-Displacements of the Uterus—By William H. Wathen, M. D., LL. D., Louisville, Ky.

Puerperal Eclampsia, or Albuminuria—By T. S. Booth, M. D., Ardmore, I. T.

Report of a Case of Fever Following Parturition—By J. K. P. Bowen, M. D., Farmington.

The Menopause—By H. T. Arvine, M. D., Greenville.

Vaginal Hysterectomy—By Prof. Crofford, Memphis, Tenn.

SECTION ON SURGERY.

The Prognosis of Malignant Tumors—By J. B. Smoot, M. D., Dallas.

Axillary Aneurysm—By Joe Largent, M. D., McKinney.

Urethral Stricture—By L. P. McQuistian, M. D., Paris.

The Treatment of Gunshot Wounds—By C. H. Sherman, M. D., Dallas.

Antisepsis—By T. O. Staples, M. D., Wylie.

The Treatment of Sprained Ankle—By M. M. Edmonson, M. D., Dallas.

Appendicitis—By John A. Wyeth, M. D., New York.

Internal Urethrotomy—By Prof. Rogers, Memphis, Tenn.

General Anesthetics—By S. J. Gano, M. D., Dallas.

Some Simple Operations in the Throat for the General Practitioner—By S. W. McJunkin, M. D., Dallas.

Report of a Case—By Henry Smith, M. D., Detroit.

Some Notes on Rectal Troubles—By E. J. Reeves, M. D., Dallas.

Report of Four Cases of Varicocele Treated by Subcutaneous Deligation—By W. J. Lane, M. D., Dallas.

Report of a Case of Cholecystotomy—By John J. Pendergrass, M. D., Lennard.

Local Anesthetics—By J. H. Florence, M. D., Dallas.

**Thirty-Fifth Quarterly Meeting** of the Austin District Medical Society will be held in the Knights of Pythias Hall, Austin, Texas, Thursday, June 25th, 1896.

Officers.—A. N. Denton, M. D., President, Austin; C. J. Forbes, M. D., First Vice-President, Johnson City; T. J. Tyner, M. D., Second Vice-President, Austin; S. E. Hudson, M. D., Secretary and Treasurer, Austin.

Censors.—W. T. Richmond, M. D., Chairman; R. P. Talley, M. D., J. W. McLaughlin, M. D., Matthew M. Smith, M. D., R. Atkinson, M. D., A. Jay Sibley, M. D.

You are invited to be present and participate in the following programme:

1. "Chronic Interstitial Nephritis," by Dr. J. Angus Gillis; discussion opened by Drs. E. M. Thomas and C. O. Weller.

2. "Treatment of Puerperal Convulsions," with report of a case, by Dr. T. M. Yett; discussion opened by Drs. Sam Cunningham and T. R. Pettway.

3. "Umbilical Hemorrhage," by Dr. H. B. Granberry; discussion opened by Drs. J. C. Anderson and J. M. Strayhorn.

4. "Tıeatment of Incomplete Abortions," by Dr. A. Nowlin; discussion opened by Drs. F. R. Martin and W. J. Mathews.

5. "Modern Treatment of Acute Inflammations of the Middle Ear," by Dr. Joseph A. Mullen; discussion opened by Drs. T. J. Tyner and H. L. Hilgartner.

6. Chronic Diffuse Nephritis Without Exudation—Report of cases," by Dr. Jo S. Wooten; discussion opened by Drs. W. T. Richmond and J. W. McLaughlin.

7. Voluntary papers and reports of cases.

A. N. Denton, President. S. E. Hudson, Secretary.

